# Psychosocial Effects of Corona Measures on Patients With Dementia, Mild Cognitive Impairment and Subjective Cognitive Decline

**DOI:** 10.3389/fpsyt.2020.585686

**Published:** 2020-10-26

**Authors:** Ingrid S. van Maurik, Els D. Bakker, Susanne van den Buuse, Freek Gillissen, Marleen van de Beek, Evelien Lemstra, Arenda Mank, Karlijn A. van den Bosch, Mardou van Leeuwenstijn, Femke H. Bouwman, Philip Scheltens, Wiesje M. van der Flier

**Affiliations:** ^1^Department of Neurology, Alzheimer Center Amsterdam, Amsterdam University Medical Center, Amsterdam Neuroscience, Vrije Universiteit Amsterdam, Amsterdam, Netherlands; ^2^Department of Epidemiology and Data Science, Amsterdam University Medical Center, Vrije Universiteit Amsterdam, Amsterdam, Netherlands; ^3^Alzheimer Nederland, Amersfoort, Netherlands

**Keywords:** COVID-19, dementia, MCI, SCD, psychosocial effects, behavioral problems, discontinuation of care

## Abstract

**Background:** The recent COVID-19 pandemic is not only a major healthcare problem in itself, but also poses enormous social challenges. Though nursing homes increasingly receive attention, the majority of people with cognitive decline and dementia live at home. We aimed to explore the psychosocial effects of corona measures in memory clinic (pre-)dementia patients and their caregivers.

**Methods:** Between April 28th and July 13th 2020, *n* = 389 patients of Alzheimer center Amsterdam [*n* = 121 symptomatic (age = 69 ± 6, 33%F, MMSE = 23 ± 5), *n* = 268 cognitively normal (age = 66 ± 8, 40% F, MMSE = 29 ± 1)] completed a survey on psychosocial effects of the corona measures. Questions related to social isolation, worries for faster cognitive decline, behavioral problems and discontinuation of care. In addition, *n* = 147 caregivers of symptomatic patients completed a similar survey with additional questions on caregiver burden.

**Results:** Social isolation was experienced by *n* = 42 (35%) symptomatic and *n* = 67 (25%) cognitively normal patients and two third of patients [*n* = 129 (66%); *n* = 58 (75%) symptomatic, *n* = 71 (61%) cognitively normal] reported that care was discontinued. Worries for faster cognitive decline were existed in symptomatic patients [*n* = 44 (44%)] and caregivers [*n* = 73 (53%)], but were also reported by a subgroup of cognitively normal patients [*n* = 27 (14%)]. Both patients [*n* = 56 (46%) symptomatic, *n* = 102 (38%) cognitively normal] and caregivers [*n* = 72 (48%)] reported an increase in psychological symptoms. More than three quarter of caregivers [*n* = 111(76%)] reported an increase in patients' behavioral problems. A higher caregiver burden was experienced by *n* = 69 (56%) of caregivers and *n* = 43 (29%) of them reported that a need for more support. Discontinuation of care (OR = 3.3 [1.3–7.9]), psychological (OR = 4.0 [1.6–9.9]) and behavioral problems (OR = 3.0 [1.0–9.0]) strongly related to experiencing a higher caregiver burden. Lastly, social isolation (OR = 3.2 [1.2–8.1]) and psychological symptoms (OR = 8.1 [2.8–23.7]) were red flags for worries for faster cognitive decline.

**Conclusion:** Not only symptomatic patients, but also cognitively normal patients express worries for faster cognitive decline and psychological symptoms. Moreover, we identified patients who are at risk of adverse outcomes of the corona measures, i.e., discontinued care, social isolation, psychological and behavioral problems. This underlines the need for health care professionals to provide ways to warrant the continuation of care and support (informal) networks surrounding patients and caregivers to mitigate the higher risk of negative psychosocial effects.

## Background

The recent COVID-19 pandemic is not only a major healthcare problem in itself, but also poses enormous societal challenges (1). People living with cognitive impairment and dementia may be doubly affected by this pandemic (2). On the one end, this population is more vulnerable for severe symptoms of the infection (3, 4). On the other hand, the issued measures (i.e., social distancing, lockdown) to combat spread of COVID-19 have great impact on the lives of these patients. There has been increasing interest for the devastating situation of dementia patients living in nursing homes (5–7), but the majority of patients with cognitive decline and dementia live at home and make use of a combination of formal and informal care. Formal care, like community care services, district nurse or day care institutions, was largely shut down, which further increased the burden on informal care, i.e., the caregiver. Moreover, the informal support network of children, neighbors, and volunteers became largely ineffective as a result of the measures. In addition, there is a large contingent of memory clinic patients who experience cognitive decline, but perform normal on cognitive testing, i.e., subjective cognitive decline (8). Also in these pre-dementia phases where patients are still cognitively normal but worried, the consequences of the corona crisis may cause an unbalance in mental health.

In times of uncertainty, staying socially connected is important. Due to social distancing and/or lockdown, many people sought for social connections online, which may be more difficult for memory clinic patients and their caregivers. As a result, feelings of loneliness, anxiety and uncertainty may have increased during the corona crisis. Furthermore, finding structure during the day is particularly difficult for individuals with cognitive impairment and the lack of daycare or other activities may result in faster cognitive decline, not only in the de stage of dementia, but also in pre-dementia stages. In turn, this may negatively affect the caregiver and deteriorate mental well-being in both the patient and caregiver.

In the current study, we aimed to evaluate the psychosocial effects of corona measures in terms of discontinued care, behavioral and psychological effects in patients with pre-dementia and their caregivers living at home. In addition, we set out to identify red flags for patients likely to be most severely affected by the corona measures.

## Methods

### Patients

Between April 28th 2020 and July 13th 2020, we invited cognitively normal and symptomatic patients to complete a self-designed corona survey from the Amsterdam Dementia Cohort (9, 10). Patients were actively enrolled in one of the following three ongoing substudies of the Amsterdam Dementia Cohort: (1) SCIENCe project (11)–all with a diagnosis of subjective cognitive decline (SCD), i.e., cognitively normal. Participants with SCD attended our memory clinic for their cognitive complaints, but performed normal on cognitive testing. (2) Patients included in the DEvELOP study—all with a diagnosis of dementia with Lewy bodies (DLB), i.e., symptomatic patients; and (3) symptomatic patients included in the follow-up study of ABIDE-PET (12, 13). ABIDE-PET was a study that included patients from an unselected memory clinic cohort, and therefore contains patients with dementia, mild cognitive impairment and SCD. We invited *n* = 916 patients of whom *n* = 389 patients completed the corona survey; *n* = 268 cognitively normal and *n* = 121 symptomatic patients.

In addition, we invited caregivers of patients in (2) DEvELOP and (3) ABIDE-PET to complete a similar survey, with additional questions on caregiver burden. As in cognitively normal patients cognitive decline is not objectified and these patients function normally in daily life, they often have no informal caregiver. Therefore, partners of cognitively normal patients were not invited to fill in the caregiver survey. In total *n* = 147 caregivers [*n* = 92 (63%) patient-caregiver dyads, *n* = 55 (47%) caregiver only] participated.

### Survey on Psychosocial Effects of Corona Measures

We developed the survey in collaboration with Alzheimer Nederland and via a bottom-up approach with expert opinions from neurologists (FB, PS) and a dementia nurse (FG). The survey consisted of questions on COVID-19 infection, discontinuation of care, social isolation and psychosocial effects. Discontinuation of care included questions on housekeeping, home aid, day care, community care services and visits to the general practitioner (GP) or hospital. Regarding psychosocial effects, the questionnaire included questions on apathy, change in sleeping behavior, loneliness, anxiety, uncertainty, depression, and worries for a possible COVID-19 infection or faster cognitive decline. The caregiver survey included questions on caregiver burden, whether the patient exhibited more behavioral problems, repetitive behavior and aggression, and questions on psychosocial effects experienced by the caregiver. The complete patient and caregiver surveys can be found in the supplemental data in Supplementary Material. Questions on discontinuation of day care and community care services were omitted in the survey that was distributed among cognitively normal patients.

### Prior Cognition and Neuropsychiatric Symptoms

Demographic data of the patients were retrieved from the Amsterdam Dementia Cohort, and included age, sex, living situation, and marital status. We also retrieved the last reported mini-mental state examination (MMSE) and behavioral and psychological symptoms of dementia as reported on the neuropsychiatric inventory (NPI) (14) and geriatric depression scale (GDS) (15).

### Statistical Analysis

We compared responders and non-responders on patient characteristics (age, sex, MMSE and diagnosis) using non-parametric tests where applicable. Descriptive statistics were used to report on the frequencies of discontinuation of care, social isolation, and psychosocial effects reported by patients and caregivers. For the analyses, answers were dichotomized into present if participants agreed or completely agreed with a statement, and absent if disagreed or completely disagreed. We used univariate logistic regression analysis to identify red flags for the presence of higher caregiver burden and worries for cognitive decline. Candidate determinants were patient characteristics (age, sex, MMSE), process measures (presence of social isolation, discontinued care) and patient or caregiver related measures [presence of psychological symptoms, neuropsychiatric problems (patients only)]. Additionally, we adjusted the analyses (ORs) for dementia subtype. All analyses were carried out in STATA SE14.

## Results

In total *n* = 916 patients were invited, of which *n* = 389 (42%) responded and *n* = 527 (58%) did not. Responders and non-responders did not differ in age or proportion of females. Responders had a higher last MMSE score (27 ± 4) compared to non-responders (24 ± 6, *p* < 0.001). Responders differed from non-responders with regard to diagnosis (*p* < 0.001), as responders were more often cognitively normal and less often dementia patients (Supplementary Table 1).

Patient and caregiver characteristics of the responders are summarized in Table 1. The mean age of symptomatic patients was 69 ± 6, *n* = 40 (33%) were female and almost all [*n* = 97 (91%)] lived with a partner. Cognitively normal patients were slightly younger (66 ± 8,) *n* = 107 (40%) were female and the majority lived with a partner [*n* = 189 (76%)]. Caregivers had a mean age of 67 ± 8, *n* = 85 (69%) was female.

**Table 1:** Patient and caregiver characteristics.

		**Patients**				**Caregivers**
		**All**	**Cognitively normal**	**Symptomatic**		
		***N* = 389**	***N* = 268**	***N* = 121**		***N* = 147**
Age	389	67 ± 8	66 ± 8	69 ± 6	125	67 ± 8
Sex, F (%)	389	147 (38%)	107 (40%)	40 (33%)	124	85 (69%)
Diagnosis of patient	389				147	
SCD		268 (69%)	268 (100%)	*NA*		*NA*
MCI		35 (9%)	*NA*	35 (29%)		24 (16%)
Dementia		86 (22%)		86 (71%)		123 (84%)
AD		43 (50%)	*NA*	43 (50%)		59 (48%)
DLB		34 (40%)	*NA*	34 (40%)		44 (36%)
Dementia		9 (10%)	*NA*	9 (10%)		20 (16%)
other						
Last MMSE	384	27 ± 4	29 ± 1	23 ± 5		
Last NPI	284	10 ± 12	9 ± 11	11 ± 12		
Last GDS	162	3.6 ± 3	4.3 ± 3	3.2 ± 3		
Living situation of patient	355				131	
Alone		69 (19%)	59 (24%)	10 (9%)		13 (10%)
With partner/family		286 (81%)	189 (76%)	97 (91%)		118 (90%)
Relation to patient					125	
Partner		*NA*	*NA*	*NA*		115 (92%)
Daughter/son		*NA*	*NA*	*NA*		5 (4%)
Other		*NA*	*NA*	*NA*		5 (4%)
Patient-caregiver dyads		*NA*	*NA*	*NA*	147	92 (63%)

*AD, Alzheimer's Disease; DLB, Dementia with Lewy bodies; GDS, Geriatric Depression Scale; MCI, Mild Cognitive Impairment; MMSE, mini-mental state examination; NPI, neuropsychiatric inventory; SCD, Subjective Cognitive Decline. n = 22 caregivers did not report on own demographic data. Time between completion of corona survey and last MMSE and was 1.00 ± 0.7 years, last NPI was 2.3 ± 0.9 and last GDS was 2.2 ± 1.1*.

Seventeen (5%) patients and *n* = 4 (3%) caregivers reported that they were probably infected with COVID-19. In four of them the infection was confirmed by the GP or Municipal Health Service.

### Social Isolation and Cognitive Decline

Social isolation was experienced by *n* = 42 (35%) symptomatic and *n* = 67 (25%) cognitively normal patients. This pertained to not seeing their friends [symptomatic: *n* = 22 (52%), cognitively normal: *n* = 40 (60%)] and family [symptomatic [*n* = 24 (57%), cognitively normal: *n* = 31 (46%)] during the COVID-19 pandemic (Supplementary Figure 1). *n* = 7 (17%)] of symptomatic patients and *n* = 7 (10%) of cognitively normal patients did not go outside at all.

Half of the caregivers [*n* = 73 (53%)] was worried for faster cognitive decline in the patient. These worries were also reported by symptomatic patients themselves [*n* = 44 (44%)] and were mentioned by a subgroup of cognitively normal patients [*n* = 27 (14%)]. More than half of caregivers reported a higher caregiver burden [*n* = 69 (56%)].

### Psychological Effects

**Figure 1:**
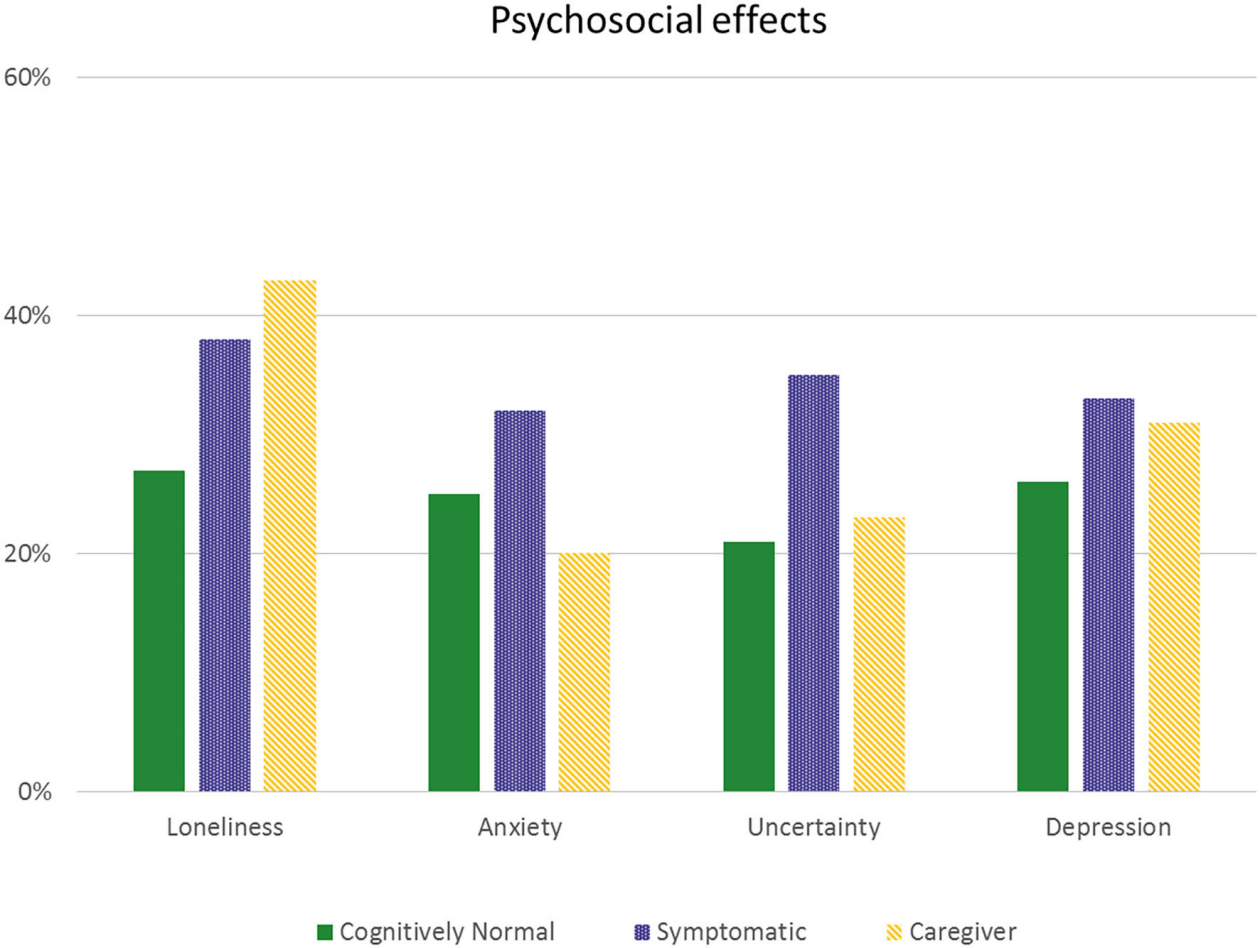
Self-reported psychosocial effects. Self-reported increase in feelings of loneliness, anxiety, uncertainty and depression in patients and caregivers.

Figure 1 presents the self-reported increase in loneliness, anxiety, uncertainty and depression by symptomatic patients and caregivers. Almost half of participants reported an increase of one or more psychological symptoms [*n* = 56 (46%) symptomatic, *n* = 102 (38%) cognitively normal and *n* = 72 (48%) caregivers].

### Behavioral Symptoms

We asked the caregivers whether they saw an increase in behavioral symptoms (apathy, changes in sleeping behavior, repetitive behavior and aggression) in the patient. An increase in patients' behavioral problems was reported by *n* = 111 (75%) of caregivers. Specifically, caregivers reported an increase in apathy [*n* = 72 (54%)], a change in sleeping behavior [*n* = 64 (48%)], increased repetitive behavior in *n* = 43 (34%) and patient aggression in *n* = 37 (30%).

When we asked patients directly about an increase in apathy and change in sleeping behavior, they reported increased apathy in *n* = 42 (40%) symptomatic and *n* = 47 (22%) cognitively normal patients. Change in sleeping behavior was reported by *n* = 40 (37%) symptomatic and *n* = 52 (25%) cognitively normal patients.

### Discontinuation of Care

**Figure 2:**
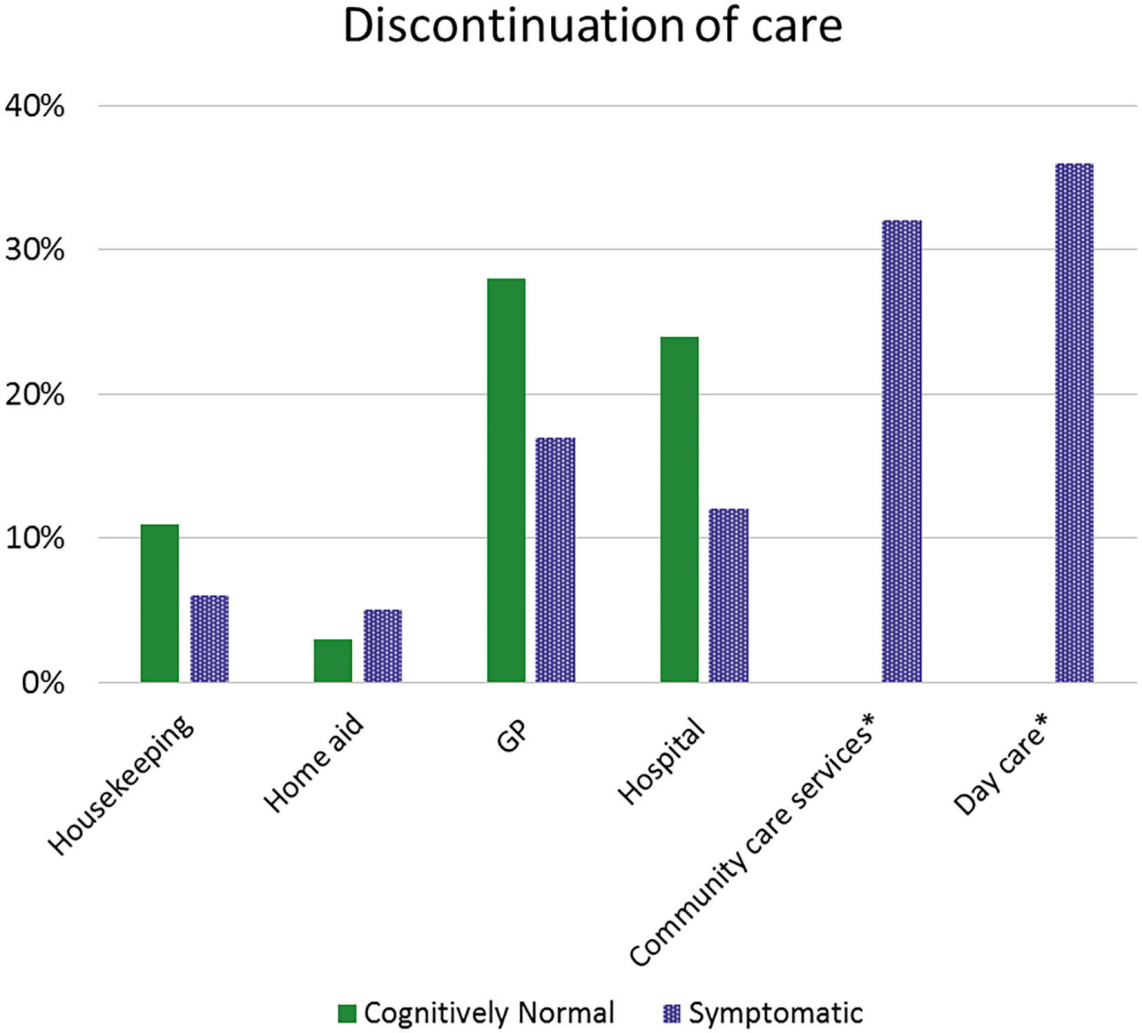
Discontinuation of care. *Discontinuation of community care services and day care were only reported by symptomatic patients. GP, General Practitioner.

*N* = 43 (36%) symptomatic and *n* = 151 (56%) cognitively normal patients did not receive any care before the COVID-19 pandemic. Of the remaining *n* = 195 (*n* = 117 cognitively normal and *n* = 78 symptomatic), *n* = 129 (66%) [*n* = 58 (75%) symptomatic, *n* = 71 (61%) cognitively normal] reported discontinuation of care (Figure 2).

Only symptomatic patients were asked on discontinuation of community care services or day care. Of those symptomatic patients, *n* = 28 (36%) reported that they were not able to go to day care and *n* = 25 (32%) reported that community care services had halted. *N* = 17 (60%) patients were offered an alternative for day care, which mostly meant contact via telephone. Of the cognitively normal patients that reported regular care from the GP, a quarter stopped visiting the GP [*n* = 33 (28%)]. Roughly one out of five [*n* = 21 (18%)] symptomatic and a small minority [14 (5%)] of cognitively normal patients indicated that they needed more support than they were currently receiving. A quarter of caregivers [*n* = 43 (29%)] reported that they needed more support.

### Red Flags

Logistic regression models were used to identify red flags for higher caregiver burden and worries for cognitive decline. Discontinued care (OR = 3.3 [1.3–7.9]), reporting one or more psychological symptoms by the caregiver (OR = 4.0 [1.6–9.9]) and behavioral problems at the patient level (OR = 3.0 [1.0–9.0]) were strongly related to a higher caregiver burden. Social isolation (OR = 3.2 [1.2–8.1]) and reporting one or more psychological symptoms by the patient (OR = 8.1 [2.8–23.7]) were determinants for worries for faster cognitive decline. Other determinants were not significant. Behavioral problems lost significance in relation to higher caregiver burden after adjustment for dementia subtype (OR = 2.3 [0.7–7.2]). Adjusting the analyses for dementia subtype did not change other results (Supplementary Table 2).

## Discussion

The current study showed that during the corona crisis social isolation, increased psychological symptoms, and discontinuation of care were frequently reported in pre-dementia patients and/or their caregivers living at home. Both patients and caregivers expressed worries for faster cognitive decline. Social isolation and psychological symptoms were red flags for these worries. Moreover, discontinuation of care, and psychological symptoms were strong predictors for experiencing a higher caregiver burden.

Social isolation due to the corona measures was experienced by one third of symptomatic patients, and by a quarter of cognitively normal memory clinic patients. Social interactions are important for patients with cognitive complaints, as is engaging in daily recreational activities, e.g., exercise (16, 17). During the corona crisis, many people sought for social connections online, but this is more difficult for patients with cognitive complaints. We even found that some patients did not go outside at all. This may worsen a patients' cognitive, mental and/or physical condition and this was indeed reported by patients (18–20). Of note, many patients were not able to go to the GP or hospital either at their own initiative or due to the closing of out-patient clinics. This may have gone at the expense of an increased risk of poor clinical outcome, also in the cognitive domain and even in cognitively normal patients, where the loss structure and social cohesion may be the final push toward onset of overt symptoms. The experience of social isolation was clearly a red flags for expedited cognitive decline and illustrates that is essential to prevent this feeling by pro-active policy aiming for social cohesion and patient empowerment, both on a government level and in the neighborhood.

The serious nature of the COVID-19 pandemic and the COVID-19 disease risk itself may also have impacted patients. As a result, the COVID-19 pandemic may have caused feelings of uncertainty and anxiety, especially in vulnerable elderly. This necessitates the availability of very easily understandable information on COVID-19. As patients with pre-dementia already lived with much uncertainty on the progression of their cognitive complaints, this may have made them more vulnerable for psychological symptoms during the COVID-19 pandemic. Due to corona (measures), half of the symptomatic patients and caregivers reported an increase in psychological symptoms, including feelings of loneliness, anxiety, depression and uncertainty. This is reason for concern as psychological and neuropsychiatric symptoms are known to be strongly related to cognitive decline, caregiver burden and quality of life (21–24). Also in cognitively normal patients, one third reported an increase in psychological symptoms. A recent review reported on the psychological impact of quarantine (25), and showed that psychological distress, amongst others depression, anxiety and insomnia, varied between 12 and 34% of people that were quarantined for several weeks (26, 27). However, these results came from the SARS epidemic in 2003, during which people were not able to go outside at all (27). In comparison, quarantine for the participants in the current study was not that stringent, as people in the Netherlands were advised to stay home, but were allowed to go outside for a walk or some grocery shopping. Nonetheless, we show that, despite these less stringent measures, psychological symptoms in pre-dementia patients and caregivers were much more frequent.

An increase in behavioral problems was reported by the three quarter of caregivers. Mostly, patients exhibited an increase in apathy or sleeping behavior, but also an increase in agitation and repetitive behavior. This may be an important moderator in the effect of discontinued care on higher caregiver burden. These behavioral problems may be even more problematic, as a recent review showed that patients who exhibit aggression, wandering or disinhibition are even at higher risk of catching and spreading COVID-19 (16), triggering a vicious circle as research now shows that catching COVID-19 has adverse impacts upon the brain and cognition.

More than half of caregivers reported a higher caregiver burden. This could even be under reported, as a recent report by the Dutch patient organization “Alzheimer Nederland” on a similar survey among caregivers, showed a higher caregiver burden in 80% of respondents (28). This difference could be due to differences in population, as the patients in our study were in general in a relatively mild disease stages. Red flags for overburdened caregivers were discontinuation of care, and the occurrence of psychological symptoms such as loneliness or anxiety either expressed by the patient or themselves. National and international efforts arise to set up conceptual frameworks that guide the management of key areas related to dementia care. In general, these frameworks point out that community-based health care professionals (HCP) together with a patients' social network play a pivotal role. Together they should identify families in need, support caregivers in dealing with problematic psychological and/or behavioral changes and help patients to engage in an active lifestyle at home. Our study shows that continuation of care is essential, and if physical visits are not possible, than alternatives, such as by phone or online should be actively pursued. Recently, in response to COVID-19 literature becomes available on how to redesign health care and telehealth has been advocated. The advantages of remote care for pre-dementia patients and their care partners may outweigh the difficulties of setting up this new way of working; outpatients do not have to visit the hospital, reduces need for traveling, minimizes complications and better fits a patients' daily routine (29, 30).

The association of psychological symptoms with higher caregiver burden and increased worries for cognitive decline shows that not only patients, but also their caregivers should be actively monitored, supported and empowered. In order to facilitate early recognition, governmental bodies should help to increase society's awareness of the challenges that vulnerable patients and their caregivers face due to corona and corona measures (31). Efforts should be made to help patients and caregivers to develop and maintain a daily routine during active lockdown measures, as the predictability of such a routine can decrease anxiety (29). Moreover, activities in and around the house can help to keep active and purposeful (20, 29). The current study adds to this by showing that there should not only be attention for symptomatic patients, but also for cognitively normal patients as they express significant worries for faster cognitive decline and often experience psychological symptoms as well.

Among the strengths of our study is the large sample of symptomatic and cognitively normal patients with different types of dementia, MCI and SCD. In addition, we had a large sample of caregivers that completed the survey. We were flexible to rise to the occasion as we had an online survey system in place in the midst of the corona crisis. As a result, we have a good overview of the effects of the corona measures on the whole spectrum of cognitive decline and dementia. While most attention has been paid to the institutionalized dementia patients, we show the vulnerability of those living at home.

Among the limitations is a potential selection bias. The included patients in the current study were able to complete a survey online, perhaps with help of a caregiver. By using an online survey we may not have reached everyone, as the survey may have been less accessible for people with severe cognitive complaints, suboptimal health literacy or diverse populations. Nonetheless, with this online nature of the survey we did befitted from the general atmosphere of the corona-times. Moreover, all patients participated in specific studies, which perhaps illustrates that they are socially active, and relatively less vulnerable. In response to the acuteness of the COVID-19 pandemic, we did not use a validated survey. Instead we developed a survey in collaboration with Alzheimer Nederland and via a bottom-up approach with expert opinions from neurologists, social scientists and dementia nurse. The survey, as any by definition, is subjective in nature and therefore we not only asked whether participants experience a certain item (for example social isolation), but also included follow-up questions to assess how this was experienced as this might differ from person to person. In addition, we did not invite partners of cognitively normal patients. In this way, we may have missed cases where cognitively normal patients did not notice that they became symptomatic, while in fact the partner did experience a sudden drop in cognitive functioning. In their patient consultations, our neurologists heard a few of such accounts. This further illustrates the relevance of awareness of the negative consequences of the corona measures particularly in pre-dementia stages.

According to simulation models, a second wave of a COVID-19 outbreak is likely to happen and new or prolonged measures to combat the spread will be issued (32). Preparing for a second wave, we show that memory clinic patients and their caregivers are a vulnerable group to look after, who experience negative impact in terms of psychological and behavioral symptoms, express worries for faster cognitive decline and experience a higher caregiver burden. This shows the need for health care providers and professionals to set up ways to warrant the continuation of care and to counsel patients and caregivers at higher risk of negative psychosocial effects.

## Data Availability Statement

The raw data supporting the conclusions of this article can be made available by the authors upon reasonable request. Requests to access the datasets should be directed to Ingrid S. van Maurik, i.vanmaurik@amsterdamumc.nl.

## Ethics Statement

The studies involving human participants were reviewed and approved by the Medical Ethics Review Committee of the VU University Medical Center. The patients/participants provided their written informed consent to participate in this study.

## Author Contributions

IM, SB, FG, FB, PS, and WF designed the study and corona survey. EB, MB, EL, AM, KB, and ML collected the data. IM conducted the data analysis and had full access to all data in the study and took responsibility for the integrity of the data and the accuracy of the data analysis. IM and WF interpreted the data and drafted the article. All authors revised the manuscript.

## Conflict of Interest

The authors declare that the research was conducted in the absence of any commercial or financial relationships that could be construed as a potential conflict of interest.
