# Supplementary material for: Psychosocial Effects of Corona Measures on Patients With Dementia, Mild Cognitive Impairment and Subjective Cognitive Decline
Source: Front Psychiatry. 2020 Oct 26;11:585686. doi: 10.3389/fpsyt.2020.585686 (PMC7649118; doi:10.3389/fpsyt.2020.585686)
Supplement: Supplementary file 1 [file Data_Sheet_1.docx]

**Supplementary Material.**

**Psychosocial effects of corona measures on patients with dementia, mild cognitive impairment and subjective cognitive decline**

Supplemental Table 1. Characteristics of (non-) responders

Supplemental Figure 1. Social Isolation

Supplemental Table 2. Odds ratio’s for adjusted logistic regression analyses

Supplemental Data 1. Corona survey for non-symptomatic patients

Supplemental Data 2. Corona survey for symptomatic patients

Supplemental Data 3. Corona survey for caregivers

**Supplemental Table 1. Characteristics of (non-) responders**

|  | **All** | **Responders** | **Non-responders** |  |
| --- | --- | --- | --- | --- |
|  | **n=916** | **n=389** | **n=527** | **p-value** |
| Age | 67±8 | 67±8 | 67±9 | NS |
| Sex, F (%) | 376 (41%) | 147 (38%) | 229 (44%) | NS |
| Diagnosis |  |  |  |  |
| SCD | 518 (57%) | 268 (69%) | 250 (47%) | <0.001 |
| MCI | 90 (10%) | 35 (9%) | 54 (10%) |  |
| Dementia | 308 (33%) | 86 (22%) | 223 (42%) |  |
| Last MMSE | 25±6 | 27±4 | 24±6 | <0.001 |
|  |  |  |  |  |

**Supplemental Figure 1. Social Isolation**


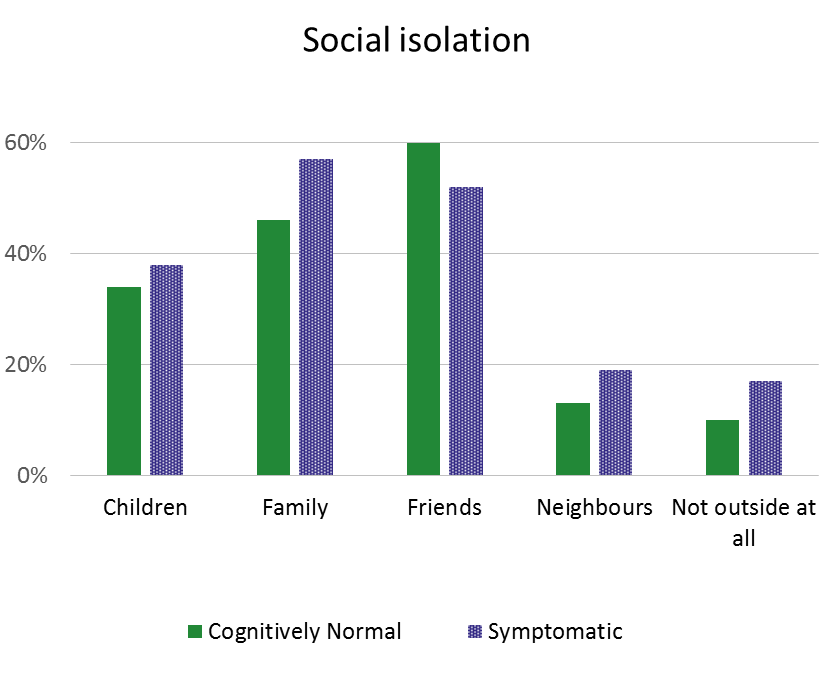


Frequencies of reduced social contacts among patients who experienced social isolation.

**Supplemental Table 2. Odds ratio’s for adjusted logistic regression analyses**

|  | ***Unadjusted*** | ***Adjusted for diagnosis*** |
| --- | --- | --- |
| ***Outcome: higher caregiver burden*** |  |  |
| Discontinued care | 3.3 [1.3-7.9] | 3.3 [1.3-8.6] |
| Psychological symptoms | 4.0 [1.6-9.9] | 4.3 [1.6-11.8] |
| Behavioral problems | 3.0 [1.0-9.0] | 2.3 [0.7-7.2] |
|  |  |  |
| ***Outcome: worries for faster cognitive decline*** |  |  |
| Social isolation | 3.2 [1.2-8.1] | 3.5 [1.3-9.4] |
| Psychological symptoms | 8.1 [2.8-23.7] | 8.2 [2.7-24.8] |

**Supplemental data 1. Corona survey for non-symptomatic patients**

| 1. Have you been infected by covid-19? | - Yes, definitely (confirmed by the GP/municipal health service) - Yes, probably - No, probably not - No, definetly not - I’m not sure | | | | | |
| --- | --- | --- | --- | --- | --- | --- |
| 1. Do you receive less care due to the corona measures than before? | - I do not receive housekeeping anymore - I do not receive home care anymore - I am no longer able to visit the general practitioner - I am no longer able to visit the hospital - Other; | | | | | |
| 1. Due to the corona measures you may experience social isolation. Is that for you the case? | - Yes - No | | | | | |
| If yes, what did social isolation encompass in your situation? | - I don’t see my children anymore - I don’t see my family anymore - I don’t see my friends anymore - I don’t see my neighbors anymore - I don’t go outside at all - Other; | | | | | |
| 1. Do you need more support than that you are currently receiving? | - Yes - No | | | | | |
| 1. To what extent do you agree with the following statements? |  | | | | | |
|  | Completely agree | Agree | Disagree | Completely disagree | I don’t know | Not applicable |
| I am less interested in my normal activities or in the activities of others | ☐ | ☐ | ☐ | ☐ | ☐ | ☐ |
| My sleeping pattern changed; for example, I get up too early or take more naps during the day. | ☐ | ☐ | ☐ | ☐ | ☐ | ☐ |
| I worry that my cognition will deteriorate faster | ☐ | ☐ | ☐ | ☐ | ☐ | ☐ |
| Since the corona crisis, I have been let down by organizations that offer me care and support | ☐ | ☐ | ☐ | ☐ | ☐ | ☐ |
| I worry that I will get infected with covid-19 myself | ☐ | ☐ | ☐ | ☐ | ☐ | ☐ |
| I feel more lonely than before, because of corona | ☐ | ☐ | ☐ | ☐ | ☐ | ☐ |
| I feel more anxious than before, because of corona | ☐ | ☐ | ☐ | ☐ | ☐ | ☐ |
| I feel more insecurity than before, because of corona | ☐ | ☐ | ☐ | ☐ | ☐ | ☐ |
| I feel more depressed than before, because of corona | ☐ | ☐ | ☐ | ☐ | ☐ | ☐ |

**Supplemental Data 2. Corona survey for symptomatic patients**

| 1. Have you been infected by covid-19? | - Yes, definitely (confirmed by the GP/municipal health service) - Yes, probably - No, probably not - No, definetly not - I’m not sure | | | | | |
| --- | --- | --- | --- | --- | --- | --- |
| 1. Do you receive less care due to the corona measures than before? | - I do not receive care for community care services anymore - I do not receive housekeeping anymore - I do not receive home care anymore - I am no longer able to visit dayc are - I am no longer able to visit the general practitioner - I am no longer able to visit the hospital - Other; | | | | | |
| 1. Were you offered an alternative for day care? | - Yes: [open text] - No - Not applicable | | | | | |
| 1. Due to the corona measures you may experience social isolation. Is that for you the case? | - Yes - No | | | | | |
| If yes, what did social isolation encompass in your situation? | - I don’t see my children anymore - I don’t see my family anymore - I don’t see my friends anymore - I don’t see my neighbors anymore - I don’t go outside - Other; | | | | | |
| 1. Do you need more support than that you are currently receiving? | - Yes - No | | | | | |
| 1. To what extent do you agree with the following statements? |  | | | | | |
|  | Completely agree | Agree | Disagree | Completely disagree | I don’t know | Not applicable |
| I am less interested in my normal activities or in the activities of others | ☐ | ☐ | ☐ | ☐ | ☐ | ☐ |
| My sleeping pattern changed; for example, I get up too early or take more naps during the day. | ☐ | ☐ | ☐ | ☐ | ☐ | ☐ |
| I worry that my cognition will deteriorate faster | ☐ | ☐ | ☐ | ☐ | ☐ | ☐ |
| Since the corona crisis, I have been let down by organizations that offer me care and support | ☐ | ☐ | ☐ | ☐ | ☐ | ☐ |
| I worry that I will get infected with covid-19 myself | ☐ | ☐ | ☐ | ☐ | ☐ | ☐ |
| I feel more lonely than before, because of corona | ☐ | ☐ | ☐ | ☐ | ☐ | ☐ |
| I feel more anxious than before, because of corona | ☐ | ☐ | ☐ | ☐ | ☐ | ☐ |
| I feel more insecurity than before, because of corona | ☐ | ☐ | ☐ | ☐ | ☐ | ☐ |
| I feel more depressed than before, because of corona | ☐ | ☐ | ☐ | ☐ | ☐ | ☐ |

**Supplemental Data 3. Corona survey for caregivers**

| 1. Have you been infected by covid-19? | - Yes, definitely (confirmed by the GP/municipal health service) - Yes, probably - No, probably not - No, definetly not - I’m not sure | | | | | |
| --- | --- | --- | --- | --- | --- | --- |
| 1. Do you need more support than you currently receive? | - Yes, for myself - Yes, for my partner - Yes, for myself ánd my partner - No, we receive sufficient support | | | | | |
| 1. To what extent do you agree with the following statements about your partner? |  | | | | | |
|  | Completely agree | Agree | Disagree | Completely disagree | I don’t know | Not applicable |
| My partner seems to have more behavioral problems |  |  |  |  |  |  |
| My partner seems less interested in his/her normal activities or in the activities of others |  |  |  |  |  |  |
| My partner refuses to cooperate or to be helped by someone else |  |  |  |  |  |  |
| My partner exhibits different sleeping behavior; for example, he/she gets up too early or takes more naps during the day. |  |  |  |  |  |  |
| My partner does activities over and over, like pace around the house without purpose, repetitive handling buttons, picking, wrapping string etc. |  |  |  |  |  |  |
| Since the coronacrisis, I have become more burdened with caring for my partner |  |  |  |  |  |  |
| I worry that the cognitive functions of my partner will deteriorate faster |  |  |  |  |  |  |
| Since the coronacrisis, I have been let down by organizations that offer me/my partner care and support |  |  |  |  |  |  |
| I worry that I will get infected with covid-19 myself |  |  |  |  |  |  |
| I worry that my partner will get infected with covid-19 |  |  |  |  |  |  |
| 1. To what extent do you agree with the following statements about yourself? |  | | | | | |
|  | Completely agree | Agree | Disagree | Completely disagree | I don’t know | Not applicable |
| I feel more lonely than before, because of corona |  |  |  |  |  |  |
| I feel more anxious than before, because of corona |  |  |  |  |  |  |
| I feel more insecurity than before, because of corona |  |  |  |  |  |  |
| I feel more depressed than before, because of corona |  |  |  |  |  |  |
